# Acceptability of the Transitional Wearable Companion “+me” in Typical Children: A Pilot Study

**DOI:** 10.3389/fpsyg.2019.00125

**Published:** 2019-02-08

**Authors:** Valerio Sperati, Beste Özcan, Laura Romano, Simone Scaffaro, Tania Moretta, Giada Turturo, Maria Nicoletta Aliberti, Vincenzo Guidetti, Gianluca Baldassarre

**Affiliations:** ^1^Istituto di Scienze e Tecnologie della Cognizione, Consiglio Nazionale delle Ricerche (ISTC-CNR), Rome, Italy; ^2^INI-Villa Dante Division, Italian Neurotraumatological Institute, Rome, Italy; ^3^Department of General Psychology, University of Padua, Padua, Italy; ^4^Section of Child and Adolescent Neuropsychiatry, Department of Human Neuroscience, Sapienza University of Rome, Rome, Italy

**Keywords:** autism-spectrum-disorder, robotics, transitional-wearable-companion, +me, therapy, social-interaction

## Abstract

This work presents the results of the first experimentation of +*me*-the first prototype of *Transitional Wearable Companion*–run on 15 typically developed (TD) children with ages between 8 and 34 months. +*me* is an interactive device that looks like a teddy bear that can be worn around the neck, has touch sensors, can emit appealing lights and sounds, and has input-output contingencies that can be regulated with a tablet via Bluetooth. The participants were engaged in social play activities involving both the device and an adult experimenter. +*me* was designed with the objective of exploiting its intrinsic allure as an attractive toy to stimulate social interactions (e.g., eye contact, turn taking, imitation, social smiles), an aspect potentially helpful in the therapy of Autism Spectrum Disorders (ASD) and other Pervasive Developmental Disorders (PDD). The main purpose of this preliminary study is to evaluate the general acceptability of the toy by TD children, observing the elicited behaviors in preparation for future experiments involving children with ASD and other PDD. First observations, based on video recording and scoring, show that +*me* stimulates good social engagement in TD children, especially when their age is higher than 24 months.

## 1. Introduction

Autism Spectrum Disorder (ASD) is a set of neurodevelopmental conditions^1^ characterized by a lifelong impairment, varying in degree, of three basic areas for the psychological development of children: limited social interaction, impaired or altered communication (both verbal and non verbal) and a restricted repertoire of activities and interests (Tsai, 1998; American Psychiatric Association, 2013). ASD can be associated with other conditions such as intellectual disabilities and epilepsy. Symptoms are usually detected in early infancy, generally around 3 years of age. However, warning signals can be detected already in the first year of life (Neimy et al., 2017) as nonresponsiveness to name, lack of spontaneous imitation, infrequent vocalizations and babbling, overfocus and perseveration on objects instead of people, minimal social smiles, or facial expressions, little or no eye contact and visual tracking, limited joint attention and social referencing, and minimal play and exploratory skills. Epidemiological data collected in several developed countries show a dramatic increase of ASD cases, from 0.7 to 1.4% of the population, in the last decades (Centers for Disease Control and Prevention, 2014; Lyall et al., 2017). Although it is not clear if such increment is due to extrinsic factors, such as the refinement of diagnostic criteria and the improved awareness about the condition (Fombonne, 2009), the relevance of the phenomenon calls for important actions for childcare support services.

There is growing consensus that early intensive interventions can have substantial benefits for children with developmental disorders (Rogers, 1996; Majnemer, 1998). This appears to be particularly true when families are involved in the rehabilitative process. In ASD treatment, parents' involvement provides additional positive therapeutic effects (an increased amount of eye contact, verbal initiations, and synchronous engagements), with collateral benefits on parents themselves (e.g., with decreased stress, increased sense of competence, higher levels of affect, Vernon et al., 2012). Early treatments seem to facilitate the acquisition and the reinforcement of pivotal social skills, possibly mitigating the severity of the condition (Dawson, 2013; Neimy et al., 2017). These observations are coherent with neurophysiological evidence showing that neural plasticity is particularly responsive at young age and when stimulated by an enriched environment and leads to both structural and functional modifications of the brain (Dawson, 2008; Calderoni et al., 2016).

The advent of new technologies, in particular digital applications, interactive robots, and computer-based toys, can be surely exploited in the field of rehabilitation as they offer new possibilities for innovative therapeutic interventions. For example, in the last 20 years there has been a considerable increase in experimental studies involving the use of *social robots* in the treatment of ASD (for some reviews, see Fong et al., 2003; Cabibihan et al., 2013; Pennisi et al., 2016). Robot companions seem to exert an effective influence on ASD children who are particularly intrigued by them. Sometimes ASD children tend to engage more with companion robots than with human partners, also exhibiting a reduction of stereotypical behaviors and increased spontaneous language production. The critical question about the long term duration of these effects is still under scrutiny and further studies are required to address it. In general, however, these results suggest that robots are a promising tool for therapies aiming to develop social skills in ASD children (Scassellati, 2007), especially if they allow a “supervised autonomy” that allows the caregivers and therapists to monitor and intervene in the child-robot interactions (Coeckelbergh et al., 2016).

Following the encouraging outcomes of *social robotics*, we developed the experimental +*me* device, the first implementation of *Transitional Wearable Companion* (TWC; Özcan et al., 2016). A TWC is a novel concept of robot characterized by three distinctive features. First, the TWC looks like a tender soft animal able to arise emotional attachment and reassuring feelings in the child, similarly to a “security blanket.” Second, the TWC is an embedded robot that responds to the child's manipulations with interesting outcomes like lights, sounds, and movements. Third, the TWC responds to the child's actions by producing “sensorimotor contingencies” for the child that can be remotely adjusted by a caregiver (a therapist or a parent) based on the child's cognitive abilities, interests, and reactions. Having these features, a TWC is a general multi-purpose device potentially usable for the therapy of PDD and ASD to enhance social, relational, and communicative skills. In particular, the TWC could be used as a medium to establish both dyadic (e.g., eye contact, imitation) and triadic (e.g., joint attention, pointing) behaviors involving the child, the robot, and the caregiver (Clifford and Dissanayake, 2009). This possibility is supported by the observation that social play activities with a high degree of immediate auditory, visual, and physical synchrony—as those furnished by toys animated by parents—provide children with rewarding social actions. This appears to be due to the fact that they are consistent, predictable, and contain physical contingency. As stated by Vernon et al. (2012, p. 2714):

“[these are] elements noted to elicit a higher degree of responding in children with autism. Through continued exposure to these motivating contingencies over time, children with autism may start to perceive social interaction to be a worthwhile endeavor and in doing so possibly modify their social developmental trajectory.”

In this work we describe a pilot study where +*me* is used for the first time in social play activities involving typically developed (TD) children with ages between 8 and 34 months and an adult caregiver. The main purpose of the experiment is to start to evaluate the potential of the proposed device and its feasibility in field research, observing and quantifying the behaviors that it elicits in typical participants. In particular we are now interested in evaluating the general acceptability of +*me* as an interesting toy able to maintain a high level of engagement in TD children, possibly also having indications on the age of highest interest. Collected data and behavioral observations will then be helpful for planning future experiments involving children with ASD and other PDD.

The rest of the paper is organized as follows. Section 2 illustrates more in detail the features of +*me*, the characteristics of the experiment participants, and the experimental protocol. Section 3 reports the results related to several behavioral indexes recorded during the tests. Finally, section 4 discusses the results and proposes future experiments involving children with ASD or similar pervasive conditions.

## 2. Materials and Methods

In this section we report the following: a brief overview of the current +*me* device, a description of the human sample along with the play activities involving the system +*me-child-adult*, and finally the procedure for data generation from video-recorded experimental sessions.

### 2.1. The +*me* Device

**Figure 1:**
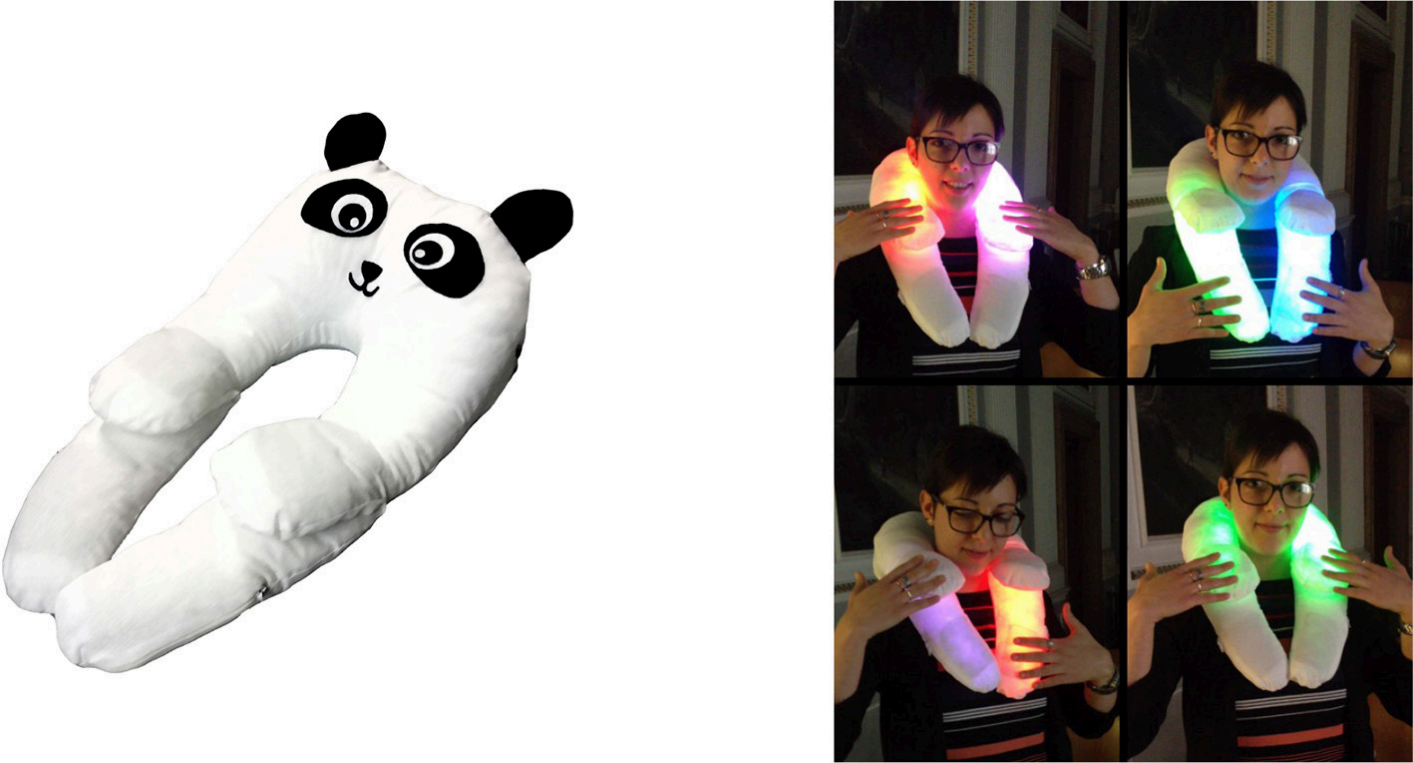
**(Left)** Current +*me* prototype used in the experiment. **(Right)** The device worn around the neck. The lighting of paws depends on the site of touch, while the color is controlled by the tablet, not present in the figure (picture published with the permission of the demonstrator).

The +*me* device looks like a soft panda with the typical features of a classic teddy bear: softness, small dimensions, big eyes. Its shape is designed to encourage “reassuring” close contact and to this purpose it can also be worn around the neck (Mullen et al., 2008; Stephenson and Carter, 2009) (see Figure 1). The technical features of the current device are described in a technical report (Sperati and Ozcan, 2016).

**Figure 2:**
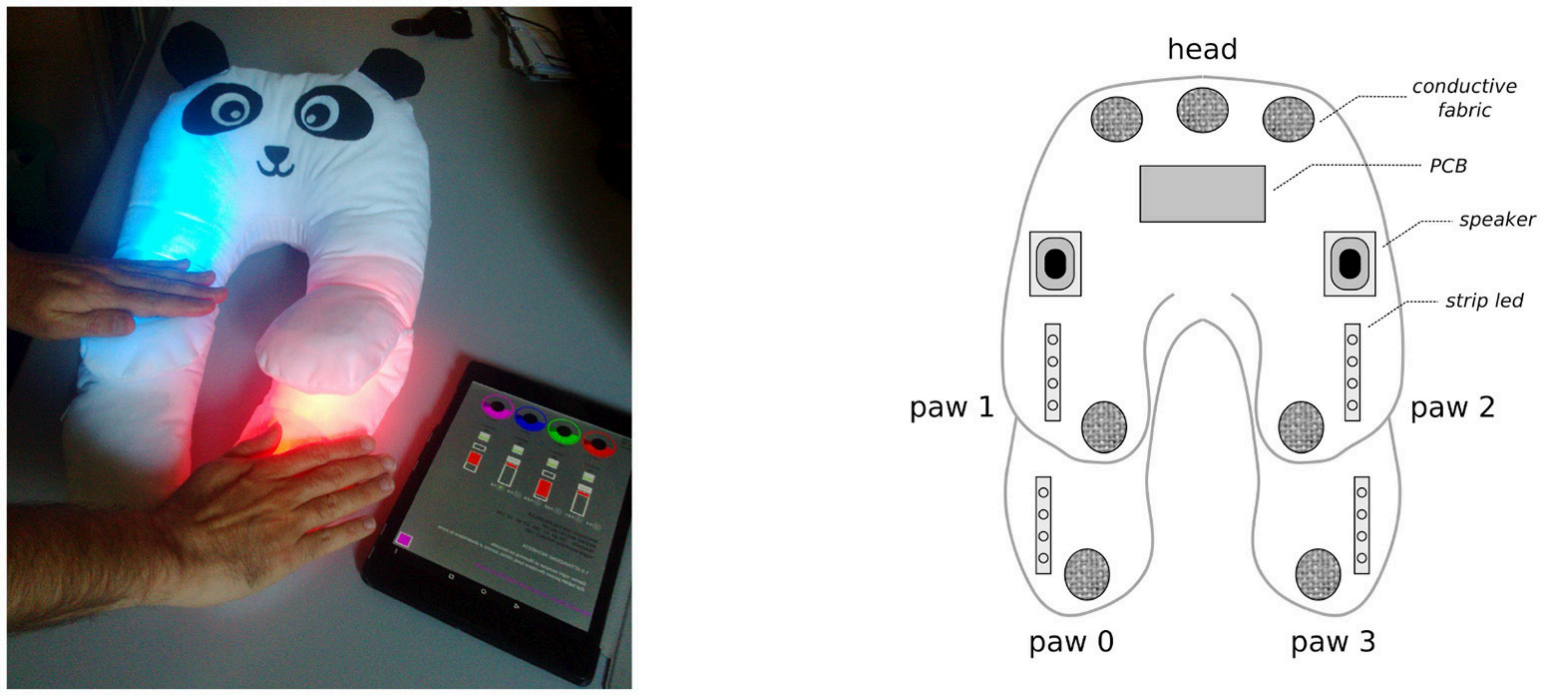
**(Left)** +*me* and the control tablet that can be used to modify the device responses. **(Right)** configuration of the inner electronic components of +*me*. Touches are detected (binary response) when the hand touches the conductive fabric patches underneath the white cotton textile; colored lights are diffused by strip LEDs embedded in the padding; sounds are emitted by speakers fixed within the panda head.

The device internally hosts dedicated Arduino-based^2^ electronics supporting the following interactions: capacitative sensors on paws and head to detect touch (binary response), and both auditory and luminous actuators (speakers and LEDs) to produce attractive responses as short amusing sounds (e.g., animal noises, the sound of a train, the tone of a bell, lasting between 2 and 5 s) and colored light sequences^3^. In the basic functioning of +*me* such responses are triggered by touching the paws of the panda. All sensory outputs can be modified by an adult through a control tablet which is connected to +*me* via Bluetooth (see Figure 2): the colors of the lights and the sounds can both be changed. Moreover, the outputs can be individually and temporarily disabled; this feature might be used by ASD therapists to adjust the level of sensory stimulation, or to stop it altogether if dysfunctional behaviors such as stereotypes are exhibited by the child. In addition to the basic functioning of +*me*, the caregiver can select more complex interactive schemes called *functions*^4^. Table 1 reports the complete list of available functions, denoted with *F*_*i*_ with *i* being the function index, and their description.

**Table 1:** Complete list of +*me* functions, selectable via tablet.

**Label**	**Effect**
**FUNCTIONS OF +*****me***	
*F*_0_	This function implements the basic functioning of +*me*, where the experimenter has the complete control of the device. The experimenter can select the paws that are responsive to touch and can select the color and sound emitted in case of touch. The available colors are red, green, blue, and magenta. The available sounds are included in a library of several mp3 files.
*F*_1_	Each paw emits a different output if touched: a brief red light on paw 0; an extended blue light on paw 1; a brief sound (harp notes) on paw 2; a phasic blue light plus a brief sound (spring noise) on paw 3.
*F*_2_	A random paw emits a blinking red light; if it is touched, a rewarding sound is emitted (trumpet notes) and the color turns to green. After a couple of seconds the game restarts with another random paw.
*F*_3_	If the +*me* head is correctly caressed (from left to right ears) the panda emits a rewarding global luminous pattern (all paws light up with different colors) and brief music (chimes notes).
*F*_4_	Soft relaxing music is played while a relaxing global luminous pattern is emitted (paws light up in blue, one after the other, continuously).
*F*_5_	If paws 1 and 2 are touched together, they light up in green and a brief sound is emitted (electronic ding).
*F*_6_	The experimenter, hitting a visible button in the app, can trigger a rewarding pattern, formed by sounds (guitar notes) and mixed colors on all paws. 4 different patterns are available.

*The tests reported here involved all functions with the exception of F_0_, which was only partially used, and F_1_, which was not used*.

**Figure 3:**
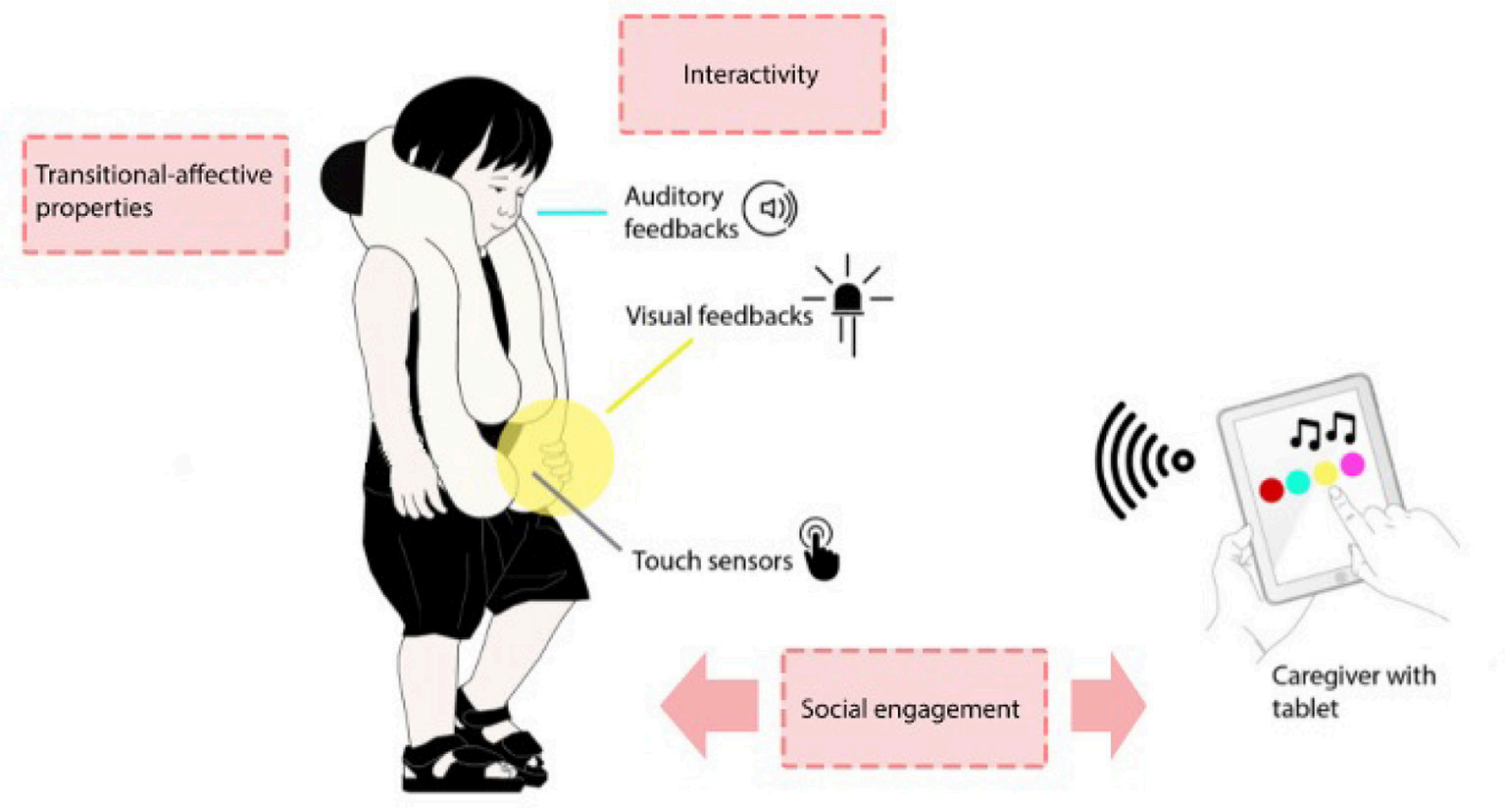
Interaction schema at the basis of the +*me* general concept. The control of the device is shared between the child, who handles the panda, and the caregiver, who handles the tablet. In a normal daily life or therapeutic context the adult can adapt the responses of +*me* (colors and sounds) according to the child's reactions and can administer enjoyable rewarding feedback when the child exhibits desirable behaviors such as cooperation (adapted by permission from Springer Nature, Özcan et al., 2016).

The concept at the basis of the +*me* device is that the triad +*me-child-caregiver* has the potential to encourage the child's development and reinforcement of social behaviors. Indeed, the features of the device are designed to attract attention and to stimulate interaction based on features eliciting intrinsic motivations (e.g., novelty, surprise, and the possibility of obtaining desirable outcomes, see Baldassarre and Mirolli, 2013). Moreover, the control of the device is *shared* between the child, who handles the panda and can act on it, and the caregiver, who can control through the tablet the sensorimotor contingencies that the device offers to the child. Crucially, this could lead the child to understand that in order to obtain a *desired, rewarding* outcome from the +*me* he/she must also engage with the caregiver (Figure 3). In the current experiment we start to explore the potential of the device by testing some of the available functions on a small group of TD children.

### 2.2. Participants and Experiment Description

We tested 15 TD children (7 females, 8 males) in a kindergarten^5^, aged between 8 and 34 months ($\bar{x}=24.5\pm 7.6$). Each child was tested in the presence of three adults, namely the experimenter (henceforth *caregiver*), the child's teacher, and an assistant in charge of video-recording the experimental session. Due to the young age of the participants, the presence of the teacher was useful to create a reassuring situation, but in no case did she actively take part in the activities between the child and caregiver^6^. This study was carried out in accordance with the recommendations of the *Ethical code of ISTC-CNR* and in particular the *Ethical Committee of ISTC-CNR* approved the experimental protocol used. Parents were informed about the purpose of the study and gave written consent to it in accordance with the *Declaration of Helsinki*.

**Figure 4:**
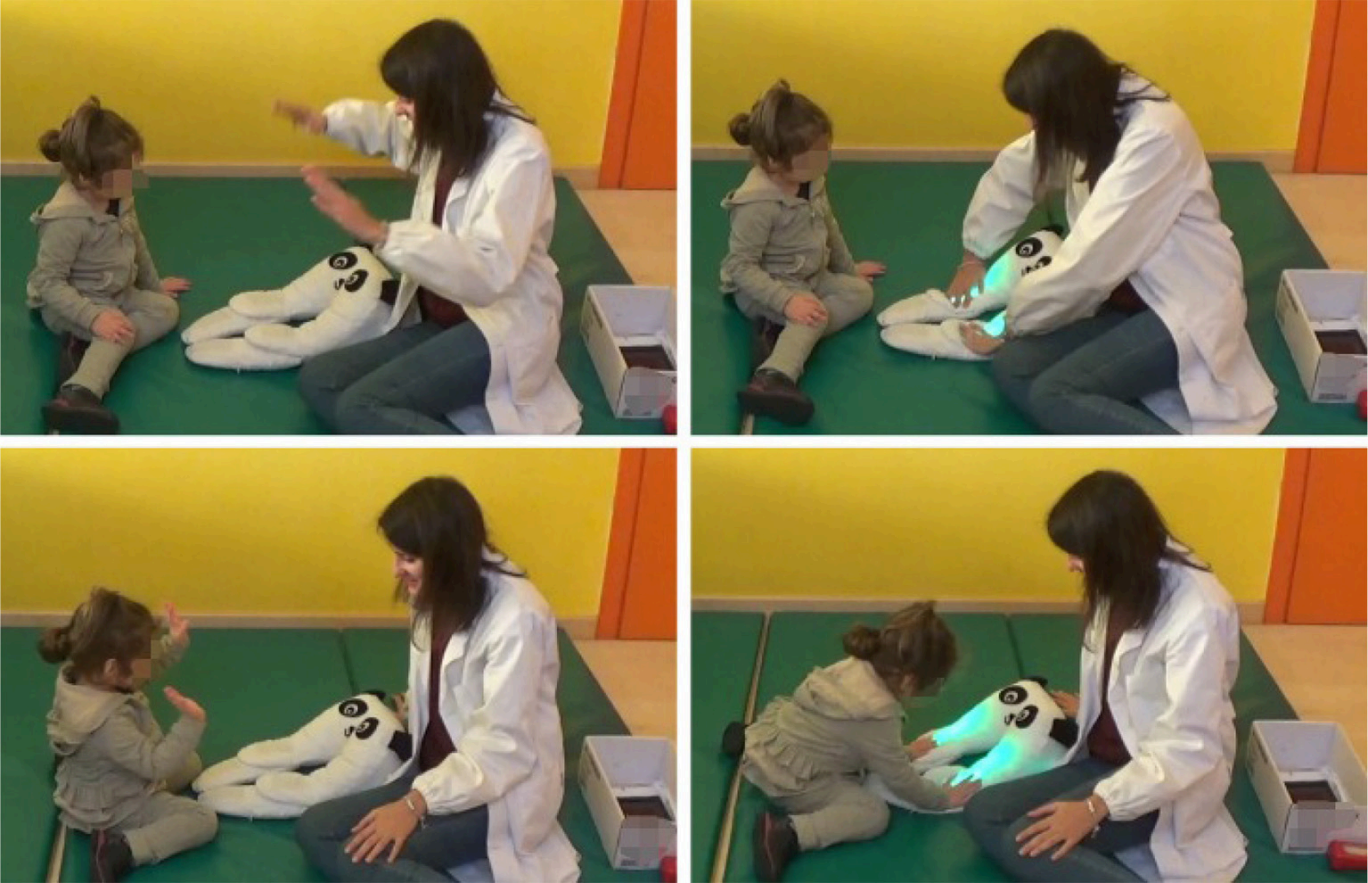
The experimenter shows a particular interaction with +*me* (activity *A*_2_ in this case), then the child (32 months) is left free to interact with the device, generally performing an imitative behavior. The tablet is hidden in the white box, in the lower-right corner of the pictures. The child's teacher and the experimenter's assistant are in the same room but do not participate in the activities (pictures published with the permission of the participant's parents and the caregiver).

The experiment consisted of 6 play activities, run in succession. Each one used a specific +*me* function and lasted about 1 min and few seconds, for a total time close to 10 min. In the setup the child sat on a carpet on the floor, the experimenter sat in front of her/him and the +*me* device was put on the floor in the middle of them. The control tablet, unless otherwise specified, was out of sight within a box close to the experimenter (see Figure 4). In order to switch from an activity to the next, the experimenter turned to the box and selected the proper +*me* function, without extracting the tablet from the box. The specific activities, denoted with *A*_*i*_ with *i* being the activity index, are now described in detail:

- *A*_1_ *(one hand imitation):* The caregiver selects function *F*_0_ on the tablet and disables paws 0, 2, and 3; then she touches paw 1, which produces a green light and the sound of a cuckoo clock (see Figure 2 for paw numbering). The action is repeated 3 times and is accompanied by encouraging expressions like “Look here!,” “What is going on here?,” “Ready? Go!.” Then she points the +*me* to the child who is left free to interact with it (the same procedure is repeated for all activities).
- *A*_2_ *(two hands imitation):* The caregiver selects function *F*_5_, then touches paws 1 and 2 with two hands and this produces green lights in the paws and a sound (see Figure 4).
- *A*_3_ *(gesture imitation):* The caregiver selects function *F*_3_, then caresses the panda head which produces a generalized rewarding pattern (lights of different colors on all paws and brief music).
- *A*_4_ *(reward game):* The caregiver selects function *F*_2_, then touches the random red-blinking paw, which produces a green light and a sound.
- *A*_5_ *(reward patterns):* The caregiver selects function *F*_6_. Then she extracts the tablet from the box, shows it to the child and triggers one of the available rewarding patterns. While doing this, she highlights her gesture to touch the tablet by saying “Look here!.” This is the only activity where the control role of the tablet is shown to the child.
- *A*_6_ *(wearability):* The caregiver selects function *F*_4_ and wears +*me* around her neck. Then she proposes that the child wears the device.

These activities aim to motivate the child to physically interact with +*me* (and tablet in *A*_5_) essentially through imitative behaviors encouraged by the caregiver. The only exception is the activity *A*_6_, which will be briefly discussed in section 3. It is important to note that the various activities all have the same purpose: to arouse the participants' curiosity and stimulate their engagement. In this respect, the experiment can be considered as a single 10-min task using the main +*me* features; indeed, the rationale behind the experimental design is to present children with an overall activity that leverages the +*me* to maintain a high interest in the interactions with the caregiver.

### 2.3. Data Generation

Based on the recorded video sessions, we computed for each child the duration and frequency of 12 different behaviors listed in Table 2, chosen as they reasonably furnish a quantitative description of the interactions involving the system +*me-child-caregiver* (see Figure 8 showing some examples). These indexes can be roughly grouped into two classes. The first class includes six indexes (from *touchP* to *smileP*) that measure the interaction between the child and +*me* and allow the assessment of the general interest raised by the device. The second class includes the last six indexes (from *smileEx* to *watchTablet*) and measures—with the exception of *cry*—the interaction between the child and the caregiver and allows the assessment of the potential benefits for the child's social abilities.

**Table 2:** List of recorded behaviors.

**Label**	**Behavior description**
**DATA RECORDS**	
touchP	Child touches +*me* (whatever contact between hand and device is recorded)
holdP	The child holds +*me* (e.g., he picks it up, hugs it or flips it)
watchP	The child looks at +*me*
refuseP	The child refuses +*me* (he shows aversion, irritation or discomfort)
move away	The child moves away from +*me* and the experimenter (he loses interest or gets distracted)
smileP	The child smiles at +*me*
smileEx	The child smiles at the experimenter
cry	The child cries
touchEx	The child touches the experimenter
watchEx	The child looks at the experimenter
pointing	The child performs a pointing behavior with the hand (to +*me* or to experimenter)
watchTablet	The child looks at the tablet (only for activity *A*_5_)

*Labels were used in the boxplots*.

The computation of the behavioral indexes was made as follows. Analyzing through slow motion (Lange-Küttner and Crichton, 1999) the recordings with standard video editing software, the experimenter computed how many seconds and how many times the child performed the selected behaviors within the 10 min experiment. Both extended behaviors (lasting more than 1 s) and phasic behaviors (lasting <1 s) were scored; in the last case the duration was considered to be 0 s long. As there is no break in the transition between the specific activities (i.e., they are run one after the other, and the duration for switching functions on the tablet is negligible) data were recorded along the whole duration of the experiment. Note that off-experiment situations (e.g., the child loses interest or gets distracted) are included in the behavior *move away*.

In order to evaluate the reliability of the collected data, an additional rater, randomly selected from a list of psychologist volunteers in research training and totally blind to the study aims, rated 30% of the recorded data (5 videos randomly selected from the set of 15). Both raters used the same scoring procedure. The inter-rater reliability (IRR) was assessed using a two-way mixed, consistency, single-measures units intra-class correlation (ICC; McGraw and Wong, 1996). The reliability was satisfactory (Cicchetti, 1994; Hallgren, 2012) for both duration (ICC = 0.80) and frequency (ICC = 0.64) across the recorded behaviors^7^. The lower ICC value for frequency is due to the fact that very brief, consecutive events (within 1 s) are sometimes difficult to be distinguished (e.g., consecutive quick touches on the same paw); in particular, we observed that rater 2 reported higher frequency values across the observed variables in comparison to rater 1, while duration evaluations were more consistent. In future research, the use of additional cameras may improve the accuracy of event detection.

## 3. Results and Discussion

**Figure 5:**
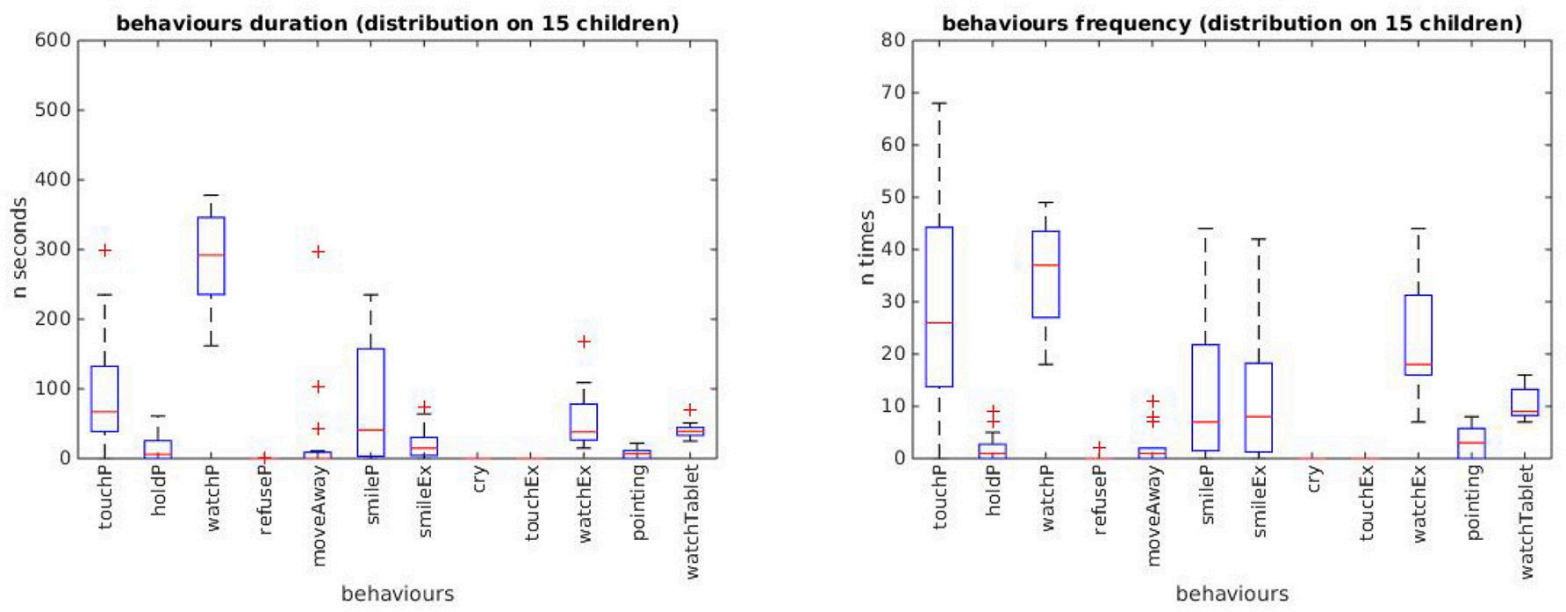
Box plots of durations **(left)** and frequencies **(right)** of the 12 behavioral indexes displayed by the 15 children during the whole experiment. For each index, the boxplot shows the level and variation of the index values: the top and bottom of each box represent respectively the quartiles *Q*_1_ and *Q*_3_, and their distance represent the interquartile range *IQR*; the middle line in the box represents the quartile *Q*_2_, i.e., the median value; the whiskers extend to the lowest and highest observations still within 1.5 × *IQR* from, respectively *Q*_1_ and *Q*_3_; crosses represent outliers beyond the whiskers.

For each behavior we obtained a distribution of the indexes' values over the sample of 15 participants as shown in Figure 5 through boxplot graphs. The whole set of boxplots furnishes an overall indication of acceptability of the device measured in terms of interactions and engagement. The “type of function” variable was not included in the analysis as the present study did not aim to test the effects of the different device functions on the behavior of the children.

A first result shown in the boxplot graphs is that children spend a certain amount of time in exploratory behaviors; they look and touch the panda, and often show enjoyment after the production of an outcome (see labels *watchP, touchP*, and *smileP*). The substantial lack of averse reactions (see labels *refuseP, moveAway*, and *cry*) suggests that the device is considered an interesting toy and manages to capture the attention of the participants.

A second result—potentially relevant for treatment of ASD and other developmental conditions characterized by social impairments—shows that children exhibit primary social behaviors toward the caregiver, such as eye contact and social smiles (see labels *watchEx* and *smileEx*), which are fundamental skills at the basis of typically developed communicative and social competence. The same behaviors manifested toward the animal-looking device (see labels *watchP* and *smileP*) mark a relevant engagement with the toy.

The comparison between the two graphs related to duration and frequency shows that the latter exhibits more distinctive values from the zero baseline value. This suggests that frequency measures are more informative than duration measures, a feature to be verified and kept into consideration in future experiments.

Qualitatively, children tend to imitate the caregiver's behavior. In particular, in the activities *A*_1_ and *A*_2_ they mostly handle +*me* paws, even if not necessarily the ones touched by the experimenter. In activity *A*_3_ they caress the panda head but also continue to touch the paws. In activity *A*_4_ the participants who “understand” the game (mostly the older ones), chase the red blinking light with the hand. In activity *A*_5_, the only one where the tablet is not hidden from sight, the tablet immediately captures the children's attention and they try to touch it to trigger the rewarding pattern (on this, we noted a strong shift of attentional focus from the panda to the tablet; this indicates that in future experiments the tablet should probably remain hidden when the tests consider it a distracting element). Direct observation of the overall children's behavior suggests that in all activities they exhibited a relevant motivation to trigger the rewarding outcomes of +*me* and showed satisfaction when they succeeded to do so (see **Figure 8** showing some examples of the observed interactions). This could be further tested in future experiments focusing on analyzing in more detail the effects of the experience of action-outcome contingencies.

In order to have the first indications about the age in which +*me* is more engaging, we divided the sample into two groups. The first group (“younger group”) was composed of 7 children aged up to 24 months ($\bar{x}=18.0\pm 5.7$) and the second group (“older group”) included the other older 8 children ($\bar{x}=30.2\pm 2.8$). Each behavioral index of the younger group was then compared with the correspondent index of the older group by running a *Wilcoxon rank-sum* test (α = 0.05, tail = left). This allowed the detection of differences between the two groups in each of the 12 indexes.

**Figure 6:**
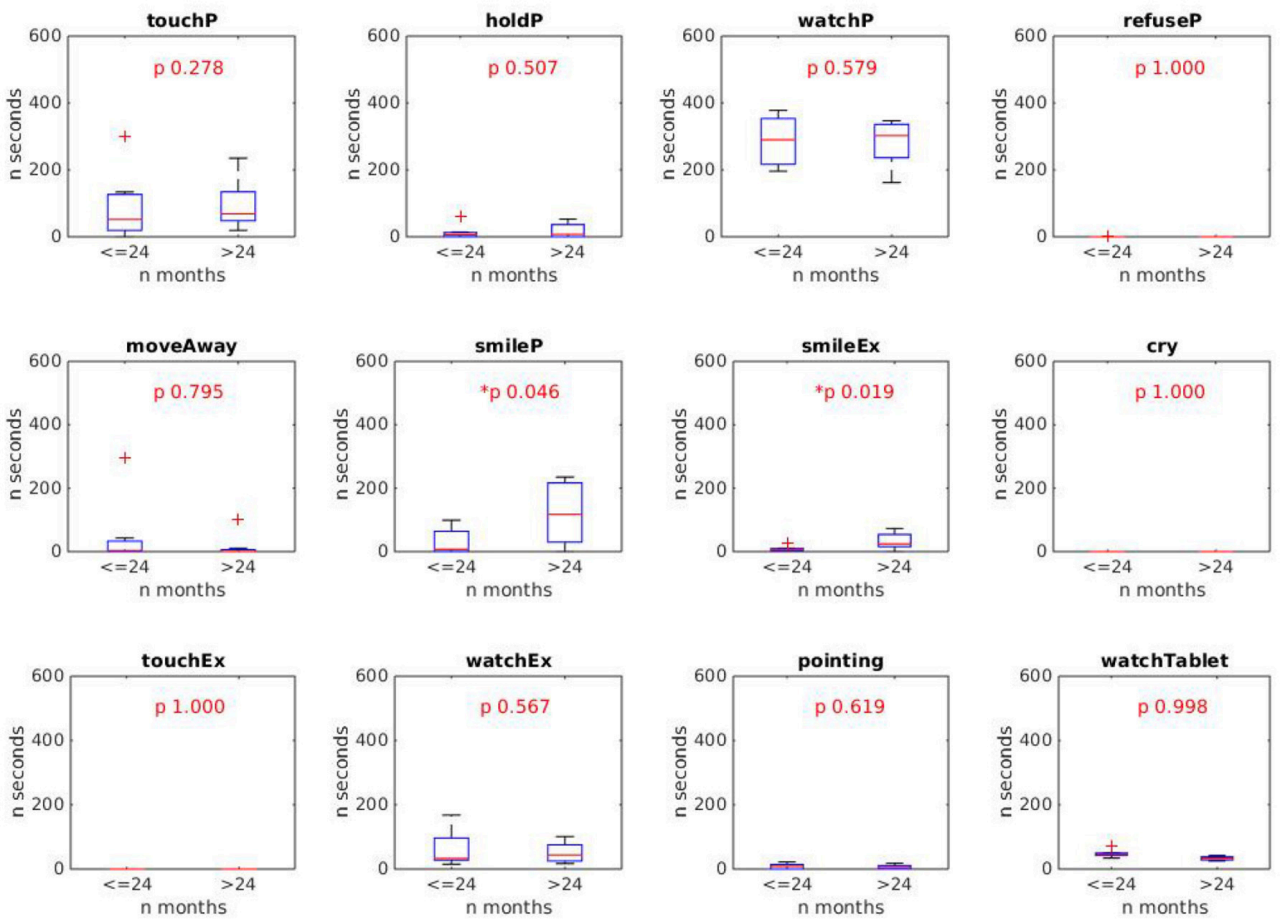
Results of *Wilcoxon rank sum* test comparing the duration of the 12 behaviors in the younger and older groups. Statistically different indexes (*p* < 0.05) are marked with the *symbol. Results show that the behaviors *smileP* and *smileEx* last longer in the older group (> 24 months).

**Figure 7:**
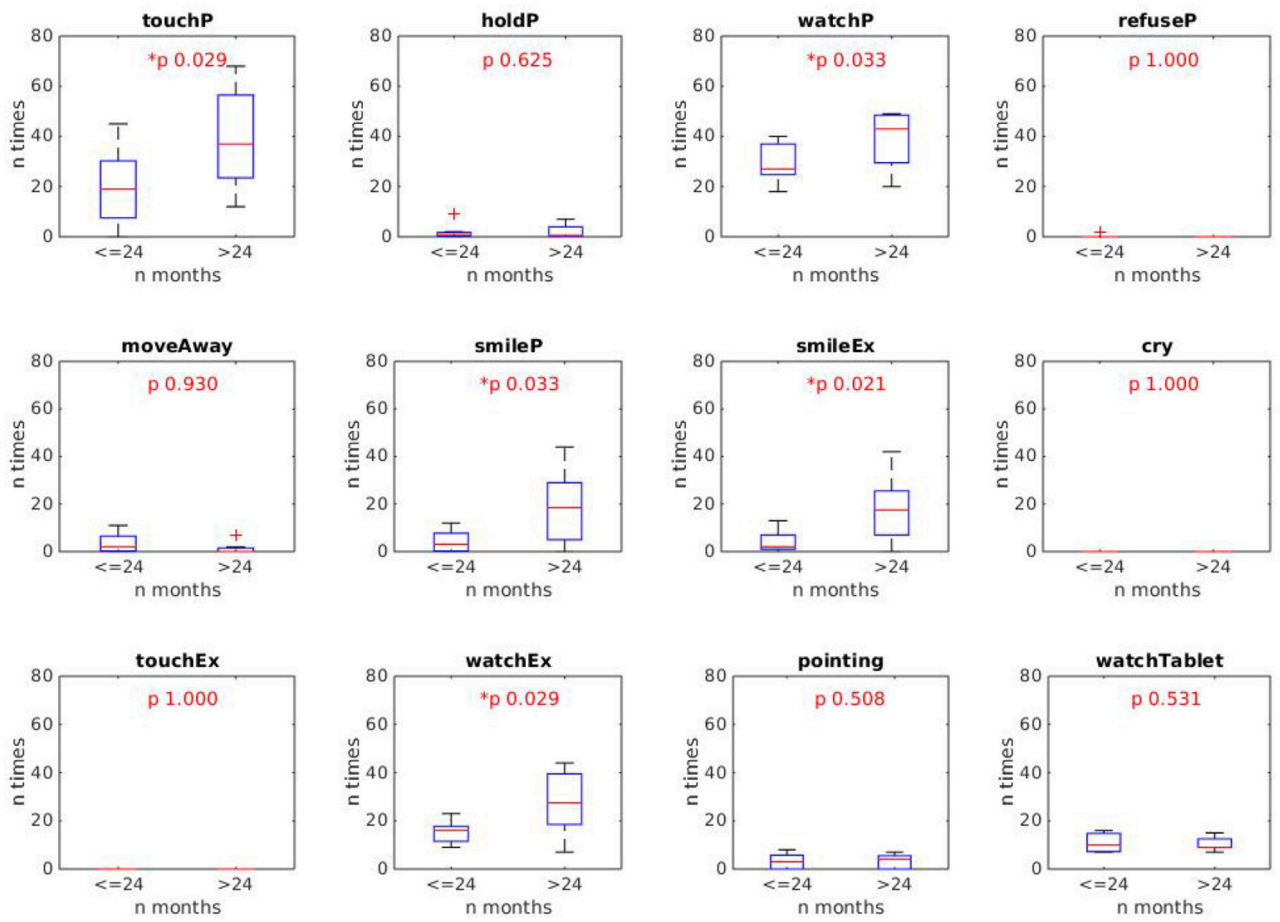
Results of the *Wilcoxon rank sum* test comparing the frequency of the 12 behaviors of the younger and older groups. Statistically different indexes (*p* < 0.05) are marked with the *symbol. Results show that the behaviors *touchP, watchP, smileP, smileEx, watchEx* are produced with a higher frequency by the older group (> 24 months).

The results, shown in Figures 6, 7, respectively for durations and frequencies, indicate a trend for which the children of the older group exhibit a greater engagement in the various activities than the children of the younger group. This result is stronger for the index frequencies (Figure 7), for which the behavioral indexes *touchP, watchP, smileP, smileEx*, and *watchEx* reveal statistically significant differences (*p-*values reported in the figure). The trend is less pronounced for the duration indexes (Figure 6), for which the behavioral indexes *smileP* and *smileEx* reveal a statistically significant difference (*p-*values reported in the figure).

**Figure 8:**
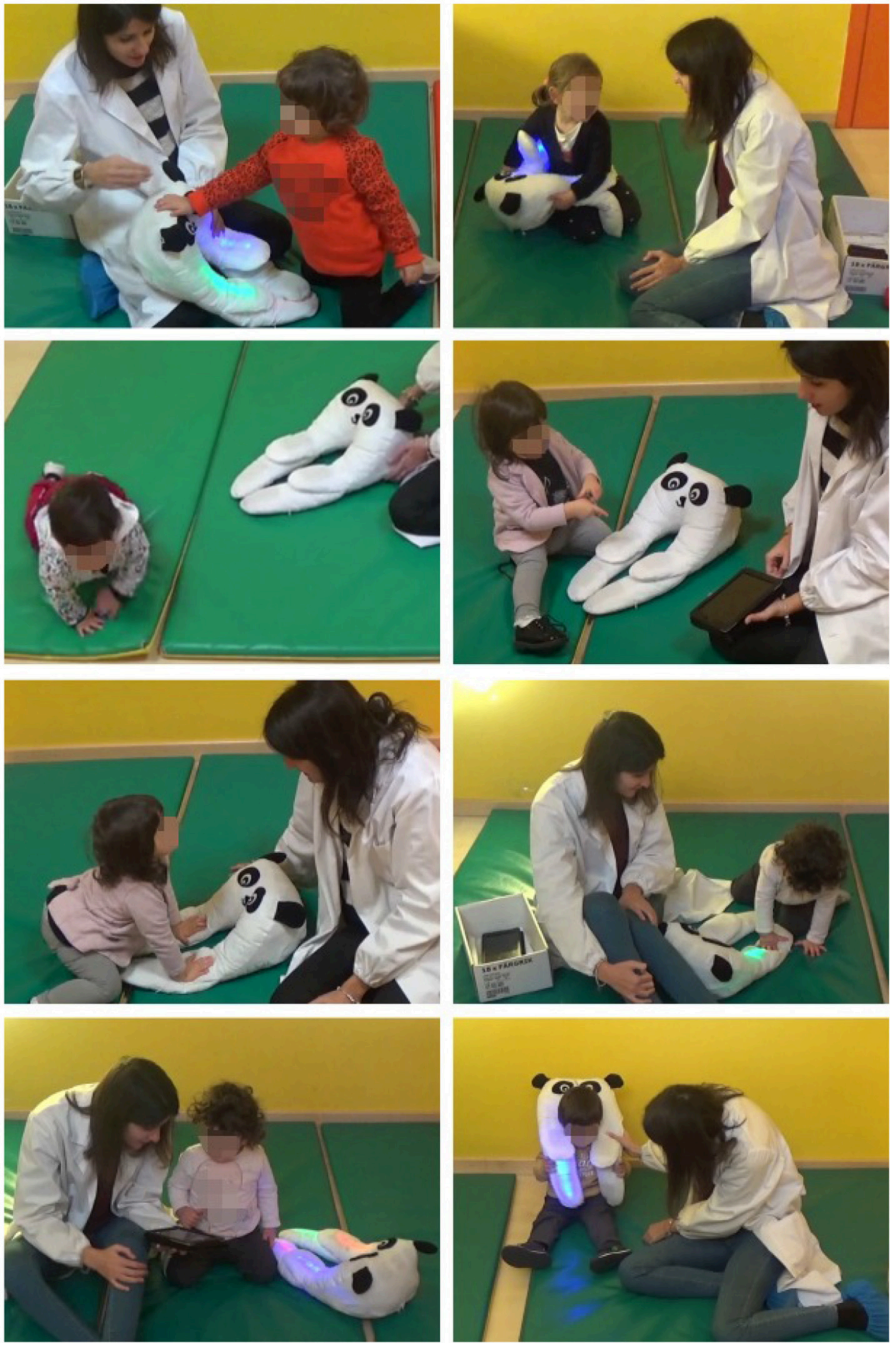
Some examples of observed behaviors. From top-left to right and bottom: touchP; holdP; move away; pointing; smileEx; touchP; watchTablet; lookEx (pictures published with the permission of the participant's parents and the caregiver).

Such a result was rather expected and can be explained by the fact that older children present greater psychological and motor development which allows them to be more responsive to the different sensorimotor contingencies offered by the +*me* device in the various activities. This was also confirmed by a qualitative inspection of the video-recorded sessions showing a greater involvement and enjoyment of children of the older group when playing with +*me* device. Nevertheless, we want to point out that even the younger children showed a certain degree of interest and sustained attention on the device (see behaviors *touchP* and *watchP* in both Figures 6, 7).

Overall, these results suggest a potential use of +*me* with participants having a psycho-motor development corresponding to an age higher than 2 years; in the terms of the article's title, these children “accept” the TWC as a toy. However, the less intense but still present interest exhibited by children of the younger group suggests that +*me* could still be useful when employed in activities proposed to younger children.

At present, we restricted the evaluation of the experimental results to a quantitative data analysis of behaviors. In particular, we reported the presence of several interesting behaviors, but we did not attempt to investigate the causal relations between the system elements, for example addressing questions like “why and when does a child smile at the panda or at the caregiver?” In order to investigate this type of relation, a more complex analysis of sensory-motor contingencies is necessary. In particular, future experiments should study behavioral sequences, for example to check if children tend to smile at the +*me* after it emits a luminous/auditory outcome and then possibly shift their attention to the experimenter. This will allow us to better understand the presence and nature of the child-caregiver social interactions possibly facilitated by the panda.

A final remark regards activity *A*_6_ on *wearability*. As mentioned in the previous section, this was the only activity in which the children were not requested to interact with the panda. This test represented a first exploration of the children's reactions when the +*me* is put on their neck. As expected, the children removed it quite quickly and put it again on the floor. However, they did not do so as an adverse reaction but rather to resume the playing activities with the panda. Plausibly this behavior was due to the novelty of the device. In this respect, the shape of +*me* facilitating its *wearability* was designed for children who, after the establishment of a sense of closeness with the device, could possibly develop an emotional attachment to the wearable object and thus actively look for reassuring, physical contact with it in daily life. This idea is supported by numerous observations about the reassuring effects of wearing weighted tight vests in children with ASD (Mullen et al., 2008; Stephenson and Carter, 2009). At the moment this property of the +*me* is only speculative and should be further investigated in further experiments. In this respect, it is worth mentioning that wearable technologies are increasingly raising interest in ASD therapy, as they have the potential to improve the quality of life of autistic individuals (Koo et al., 2018).

## 4. Conclusions and Future Work

A common challenge in the treatment of children with developmental disorders characterized by social impairments, as in Autism Spectrum Disorder (ASD), is how to sustain their attention and engagement motivations to improve their social skills (Tennyson et al., 2016). Several experimental studies have shown the potential efficacy of using robots as social catalysts when used as therapeutic tools, in particular that such artificial agents stimulate the interest of children with ASD and elicit exploration and interaction behaviors. It has been hypothesized that the reason of these effects is that the high variability of the interactions with other humans might be difficult to process and manage by individuals with ASD, whereas this variability is much reduced in the relationship with social robots that tend to exhibit simpler—but still interesting—and more predictable behaviors (Pennazio, 2017). Moreover, treatments using robots as social mediators allow the therapists to set up activities oriented to stimulate social interactions, in particular joint attention, turn taking, imitation, communication, and the accomplishment of shared goals. These elements are also present in a variety of evidence-based interventions enhancing social skills, for example in Applied Behavior Analysis (ABA), used to develop the social competencies of individuals with ASD (Tennyson et al., 2016).

The +*me* device is an experimental robotic tool that falls in the framework of *social robotics*. Its shape, texture, and sensorial outcomes are designed to stimulate at suitable levels the visual, auditory and tactile sensory channels of toddlers. An additional critical feature of the device is the possibility of controlling it in a shared fashion by the child and the caregiver (this was only partially explored in the present work): this allows the design and implementation of play activities involving rich social interaction. Overall, these features of +*me* have notable potential to attract the attention and increase the motivation and the social engagement of children. For these reasons, +*me* represents a potential useful tool for therapy of ASD and other developmental disorders impairing social abilities.

In this paper we presented the preliminary results of the first test of +*me* on a group of TD children aged between 8 and 34 months. The main purpose of the study was to assess the general acceptability of the tool as an interesting toy during an overall 10-min play activity involving an adult caregiver. Quantitative behavioral data, collected through video-recorded sessions, suggest that—on average—toddlers find it interesting to interact with the device. This indicates that the +*me* can indeed be used as a stimulating toy.

The engagement with +*me* was more marked for participants older than 24 months, but still present—even if less intense—in younger children. The general interest toward the device and the activities proposed by the caregiver were revealed by the frequency and duration of important behaviors such as gaze direction, smile, pointing, and physical contact with the device and in the interaction with the caregiver. As these behaviors (especially eye-contact and social smiles) are very important for social interaction, the results are potentially relevant for ASD treatment and other developmental disorders.

We are aware that our conclusions should be interpreted in the light of some methodological limitations of this pilot experiment. We list here the most important ones to be addressed in future experiments. First, the current results rely on the observation of a small sample of participants; future experiments should test larger groups to have stronger statistical evidence. Second, in order to better evaluate the utility of the device as an effective tool to foster interaction, one or more control conditions using comparable toys lacking some of the features of +*me* should be tested. Last, the present data only show the presence and variability of some children's behaviors. More complex analyses could instead investigate the causal relations between the behaviors of the children, +*me* and the caregiver. In particular, future experiments should study more in depth the temporal contingencies between the child's actions and the +*me* outcomes, as they are probably very important to explaining the observed behaviors.

Given these limitations, but also the encouraging results on the acceptability and the capacity of the device to stimulate engagement and social interactions, we envisage a set of possible experiments that might be run in the future:

- The same experimental protocol used here should be used to test a comparable group of participants with ASD or similar pervasive conditions. If an engagement comparable with the one of typically developed children is observed, this would encourage the use of the +*me* in therapy (of course preceded by the design of new activities appropriate for the specific conditions). The less intense but still present interest observed in younger participants requires further investigations: in this respect, we report preliminary, interesting observations by developmental therapists who informally watched the videos of the experiment, who envisaged a possible use of the device even with low-functioning ASD children, or children with other developmental disorders, where the mental development age could be <24 months.
- It would be interesting to test again the same participants of this study to track the temporal effects of +*me* across multiple training sessions. This could reveal the efficacy of the device in fostering robust long-lasting bonds with the device itself and the development of social competencies.
- It would be interesting to observe the children's engagement in an unstructured experimental context where the caregiver is free to adapt the +*me* responses (e.g., the colors and sounds, or the selection of a favorite function) according to the child's requests and reactions (see Figure 3). This aspect was not investigated in the current work as the caregiver had to follow the rigid experimental procedure. We believe that such a type of experiment would test the critical feature of the shared controllability of the device and its potential effect in fostering social interactions.
- The device should be tested with typically developed children older than 34 months to ascertain the age at which it becomes no more interesting as it is regarded as too simple. On this we indeed expect that +*me* remains attractive only for younger children.

A further point we want to stress is the potential versatility of the +*me* device. Considering that the hardware is fixed (LEDs, speakers, sensors), it is clear that the main complexity lies in the software of the control tablet, which currently implements only 7 functions (see Table 1). In this respect, it would be useful to develop software (e.g., an app) that allows the caregiver/therapist to build new interactive games (functions) through a user-friendly interface by setting novel action-outcome contingencies, activities, and rewarding patterns.

If the concept of +*me* will reveal itself as effective in fostering social interaction, then the device could be used as a tool to support therapeutic interventions involving professional therapists and possibly parents. The latter possibility, made possible by the facility of control of +*me* via standard tablets or smartphones, might represent a valuable feature of the device. Indeed, involving parents in rehabilitative processes provides an additional positive effect on children and also positive benefits on parents themselves (decreased stress, increased sense of competence, higher levels of affect; Vernon et al., 2012).

## Author Contributions

The manuscript was written by VS, who also developed the hardware and control software of the device. +*me* shape was designed by BÖ, who assembled the current prototype and also developed the original concept of Transitional Wearable Companion. The experiment with children was run by LR, assisted by BÖ, SS, and VS. Data analysis was made by VS, LR, TM, and GT. The experimental design was conceived by TM, SS, LR, and GB. The project was supervised by GB (for ISTC-CNR), MA (for INI-Villa Dante Division), and VG (for University of Rome Sapienza). The manuscript was revised by all authors.

### Conflict of Interest Statement

The authors declare that the research was conducted in the absence of any commercial or financial relationships that could be construed as a potential conflict of interest.

## Footnotes

^1^According to DSM-5, ASD groups together Autism, Asperger's syndrome, and Pervasive Developmental Disorders Not Otherwise Specified—PDD-NOS: these together represent the majority of Pervasive Developmental Disorders.

^2^Arduino is an open-source electronic platform, see official website www.arduino.cc/.

^3^The current prototype does not yet support robotic movements of paws and ears.

^4^Video material can be found at this link: https://vimeo.com/259130096.

^5^Two additional children were not included in data analysis as they completely refused any interaction with both the caregiver and the device.

^6^The teacher stayed silent in a corner of the room, near the assistant.

^7^Higher ICC values indicate greater IRR, with an ICC estimate of 1 indicating perfect agreement and 0 indicating only random agreement (Hallgren, 2012).
